# Dietary exposure to acrylamide and breast cancer risk: results from the NutriNet-Santé cohort

**DOI:** 10.1093/eurpub/ckac130.090

**Published:** 2022-10-25

**Authors:** A Bellicha, G Wendeu-Foyet, X Coumoul, M Koual, F Pierre, C Debras, B Srour, E Kesse-Guyot, M Deschasaux-Tanguy, M Touvier

**Affiliations:** 1 University Sorbonne Paris Nord, Nutritional Epidemiology Research Team, Bobigny, France; 2 Nutrition And Cancer Research Network, Paris, France; 3 Université Paris Cité, INSERM UMR-S1124, T3S, Paris, France; 4 Hôpital Européen Georges-Pompidou, Chirurgie Cancérologique Gynéco. et du sein, Paris, France; 5 Toulouse University, Toxalim - Research Centre in Food Toxicology, Toulouse, France

## Abstract

**Background:**

Acrylamide is classified as a probable human carcinogen by the IARC but epidemiological evidence on the carcinogenicity of acrylamide from dietary sources is limited. This study aimed to investigate the associations between dietary acrylamide and breast cancer risk in the NutriNet-Santé cohort.

**Methods:**

This prospective cohort study included 80,597 French women (mean [SD] age at baseline: 40.8 [14] years) during a mean (SD) follow-up of 8.8 (2.3) years. Acrylamide intake was evaluated using repeated 24h dietary records (n = 5.5 [SD 3.0]), linked to a comprehensive food composition database. Associations between acrylamide intake and breast cancer risk (overall, premenopausal and post-menopausal) were assessed by Cox hazard models adjusted for known risk factors.

**Results:**

The mean (SD) dietary acrylamide intake was 30.1 (21.9) µg/d (main contributors: coffee, potato fries and chips, pastries and cakes, and bread). During follow-up, 1016 first incident breast cancer cases were diagnosed (431 premenopausal, 585 postmenopausal). A borderline significant positive association was observed between acrylamide intake and breast cancer risk overall (HRQ4 vs Q1= 1.21 [95% CI: 1.00-1.47]) and a positive association was observed with premenopausal cancer (HRQ4 vs Q1= 1.40 [95% CI: 1.04-1.88]). Restricted cubic spline analyses suggested evidence for non-linearity of these associations, with higher HR for intermediate (Q2) and high (Q4) exposures. Receptor-specific analyses revealed a positive association with estrogen receptor-positive breast cancer, which represented 86% of total cancer cases. Acrylamide intake was not associated with post-menopausal breast cancer.

**Conclusions:**

Results from this large prospective cohort study suggest the potential deleterious role of dietary acrylamide in breast cancer etiology, especially in premenopausal women, and provide new insights that should encourage further mitigation strategies to reduce the content of acrylamide in food.

**Key messages:**

- Results from this large prospective cohort study suggest that dietary acrylamide exposure is associated with an increased risk of breast cancer, especially in premenopausal women.
- These epidemiological findings are in line with experimental data that conducted IARC, EFSA and other public health institutions worldwide to classify acrylamide as a probable carcinogen for humans.

